# Impact of high dose of baricitinib in severe COVID-19 pneumonia: a prospective cohort study in Bangladesh

**DOI:** 10.1186/s12879-021-06119-2

**Published:** 2021-05-07

**Authors:** Md. Jahidul Hasan, Raihan Rabbani, Ahmad Mursel Anam, Shihan Mahmud Redwanul Huq, Mohammad Mufizul Islam Polash, Shahzadi Sayeeda Tun Nessa, Sitesh C. Bachar

**Affiliations:** 1 Clinical Pharmacist (Critical Care and Infectious Diseases/ Stewardship), Clinical Pharmacy Services, Department of Pharmacy, Square Hospitals Ltd., 18/F Bir Uttam Qazi Nuruzzaman Sarak, West Panthapath, Dhaka, 1205 Bangladesh; 2 Internal Medicine and Intensive Care Unit, Department of Medical Services, Square Hospitals Ltd., 18/F Bir Uttam Qazi Nuruzzaman Sarak, West Panthapath, Dhaka, 1205 Bangladesh; 3 High Dependency Unit (HDU), Department of Medical Services, Square Hospitals Ltd., 18/F Bir Uttam Qazi Nuruzzaman Sarak, West Panthapath, Dhaka, 1205 Bangladesh; 4 Intensive Care Unit, Department of Medical Services, Square Hospitals Ltd., 18/F Bir Uttam Qazi Nuruzzaman Sarak, West Panthapath, Dhaka, 1205 Bangladesh; 5 Department of Pharmacy, University of Dhaka, Dhaka, Bangladesh

**Keywords:** Baricitinib, Severe COVID-19 pneumonia, Cytokine storm, High dose, Usual dose

## Abstract

**Purpose:**

Hyperinflammation in severe COVID-19 infection increases the risk of respiratory failure and one of the cogent reasons of mortality associated with COVID-19. Baricitinib, a janus kinases inhibitor, can potentially suppress inflammatory cascades in severe COVID-19 pneumonia.

**Methods:**

The objective of this study was to compare the clinical outcomes of high dose of baricitinib with its usual dose in patients with severe COVID-19 pneumonia. This prospective cohort study was conducted on 238 adult patients with severe COVID-19 pneumonia. Eight milligram and 4 mg of baricitinib was given orally to 122 patients in the high dose (HD) group and 116 patients the usual dose (UD) group, respectively daily for 14 days, and clinical outcomes were compared among the groups.

**Results:**

Blood oxygen saturation level was stabilized (≥94% on room air) earlier in the HD group compared to the UD group [5 (IQR: 4–5)/8 (IQR: 6–9), *P* < 0.05]. Patients in the HD group required intensive care unit (ICU) and intubation supports more in the UD group than that in patients of the HD group [17.2%/9%, *P* < 0.05; 11.2%/4.1%, *P* > 0.05; *N* = 116/122, respectively]. The 30-day mortality and 60-day rehospitalization rate were higher in the UD group than the HD group [6%/3.3%, *P* < 0.01; 11.9%/7.6%, *P* > 0.05; *N* = 116/122, respectively].

**Conclusion:**

The daily high dose of baricitinib in severe COVID-19 results in early stabilization of the respiratory functions, declined requirements of critical care supports, reduced rehospitalization with mortality rate compared to its daily usual dose.

## Background

The first outbreak of novel coronavirus disease 2019 (COVID-19) caused by severe acute respiratory syndrome coronavirus 2 (SARS-CoV-2), a single-stranded RNA virus, was expedited from the city of Wuhan, China in December 2019 [1, 2]. In January 2020, the World Health Organization (WHO) revealed the outbreak of a global health emergency, and in March 2020, WHO declared COVID-19 a pandemic infectious disease [2]. As of 22 November 2020, over 57.8 million confirmed COVID-19 cases and 1.3 million deaths reported across the world [3].

The outbreak first came under the spotlight as an unusual viral pneumonia and till to date, atypical upper respiratory tract pneumonia is the major feature of the severity of the disease [4]. This respiratory virus binds to angiotensin-converting enzyme 2 (ACE2) receptors by using its spike proteins to enter into the host cells. The entry of SARS-CoV-2 may result in the development of cytokine storm in the host body characterized by high plasma level of pro-inflammatory cytokines, including interleukin (IL)-6, IL-2, IL-7, IL-10, monocyte chemoattractant protein (MCP1), macrophage inflammatory protein (MIP1A), TNF (tumor necrosis factor)-alpha, and interferon gamma inducible protein (IP10) [5, 6]. The bronchoalveolar lavage taken from the patients with severe COVID-19 pneumonia showed high level of chemokines secreted from the macrophages [7]. Post-mortem analysis of lung tissue of patients with severe COVID-19 pneumonia also found excessive amount of immune cell infiltration [8]. The up-regulation pattern of systemic cytokines in patients with severe COVID-19 pneumonia was found very similar to that in cytokine release syndromes, including macrophage activation syndrome, manifested by elevated level of cytokines, such as IL-6, IL-7, and TNF-alpha, and inflammatory chemokines like, CC-chemokine ligand 2 (CCL-2), CCL-3 and CXC-chemokine ligand 10 (CXCL-10), and the soluble form of the α-chain of the IL-2 receptor, resulting in the dysregulated activation of the mononuclear phagocyte (MNP) compartment leading to hyperinflammation in patients with COVID-19 pneumonia [9].

The United States’ Food and Drug Administration (FDA) approved baricitinib for the treatment of active rheumatoid arthritis has recently been identified as a new hope for the treatment of COVID-19 pneumonia, and its use at a dose of 4 mg in COVID-19 has received the Emergency Use Authorization (EUA) from FDA on November 19, 2020 [10]. Baricitinib is a Janus kinases (JAK)-1 and JAK-2 inhibitor which exhibits dual role in the inhibition of hyperinflammation in COVID-19 pneumonia including, the inhibition of proinflammatory mediators release and endocytosis of the virus [11]. The cell-mediated signaling pathway between JAKs and signal transducers and activators of transcription proteins (STATs) is essential to activate phosphorylation process leading to cytokine release. Baricitinib inhibits this pathway selectively by blocking JAK-1 and-2 which results is down-regulation of cytokine storm in COVID-19 [11]. Recently, the Adaptive COVID-19 Treatment Trial 2 (ACTT-2) found that compared to placebo, baricitinib combinedly with remdesivir in patients with COVID-19 revealed faster recovery time [95% confidence interval (CI), 7 to 9 day versus 6 to 8 day] and reduced 28-day mortality rate (7.8 and 5.1%, respectively), significantly [12]. Another recent case-control study found that an additional 8 mg oral loading dose of baticitinib followed by 4 mg daily revealed better clinical outcomes in patients with severe COVID-19 pneumonia compared to 4 mg baricitinib daily without loading dose [13]. Though there is no standard drug has yet been developed to manage the hyperinflammation in COVID-19 patients so, experimental high dosing strategy of the available drugs, including baricitinib with assuring drug safety may be effective at this crisis moment [14]. The primary objectives of this study were to evaluate the impact of high dose versus usual dose of baricitinib on the progression of the disease, normalization of breathing function, and reduction in demand of complementary oxygen. The secondary objectives were to compare the requirement of ICU support, 60-day rehospitalization after discharge, and 30-day mortality among the patients treated with high dose or usual dose of baricitinib.

## Materials and methods

### Study design and data collection

This prospective cohort study was conducted in the “Specialized COVID-19 Unit (SCU)” of Square Hospital Ltd., Dhaka, Bangladesh (a tertiary care 400-beded private hospital), on 238 adult patients (≥18 years) with severe COVID-19 pneumonia admitted to this hospital from July 1 to October 1, 2020 directly from home. The 238 patients were selected for the study (based on the inclusion and exclusion criteria) from the total 472 hospital admitted patients with confirmed COVID-19 to COVID-19 special unit of the hospital.

All the COVID-19 positive patients were passed through a two-step triage system in the emergency department of the hospital before getting their admission in the SCU and their COVID-19 infection was confirmed by positive reverse transcriptase polymerase chain reaction (RT-PCR) assay (instrument/device: Rotor Gene-Q/Cobas z480, and QIAGEN kits for real-time PCR, QIAGEN GmbH, Germany) of two separate specimens (nasal and oral swabs) in the Molecular laboratory of the hospital. Every patient’s clinical diagnosis, comorbidities, and lab investigations were evaluated and recorded at the time of hospital admission.

All the data of patients and medications of this study were collected from the electronic patient database of the hospital and manually from the patients’ prescriptions. A dedicated multidisciplinary team consisting of four doctors and two clinical pharmacists was charged for all the patients’ data collection and drug-associated adverse event monitoring. Lab investigations and physical assessment of all the patients were performed routinely. The research related to human use has been complied with all the relevant national regulations, institutional policies, and in accordance with the tenets of the Declaration of Helsinki, and has been approved by the Research Ethics Committee, Square Hospitals Ltd., Dhaka, Bangladesh (no. 2006SH-OR027) on June 10, 2020.Written consent was taken from all participants in this study.

### Study groups and treatment

Among the 238 patients, 122 (N) and 116 (N) patients were included into the “high dose group” (HD group) and “usual dose group” (UD group), respectively using the simple random sampling method. The HD and UD group’s patients received 8 mg (into two divided doses) and 4 mg of baricitinib orally daily, respectively started within 4 h of hospital admission and continued for 14 days. Dexamethasone (corticosteroid) intravenously was given to all patients of both the groups along with the baricitinib at a dose of 0.25 mg/Kg of body weight once daily. Both baricitinib and dexamethasone was started at a time within 2 h of hospitalization in every patient. The source of baricitinib was: “Baritor 2” (baricitinib 2 mg film-coated tablet) by Square Pharmaceuticals Ltd., Dhaka, Bangladesh. Patients received intravenous remdesivir (antiviral) (200 mg loading followed by 100 mg once daily) either for 5 days (while the patient was not intubated or on bi-level positive airway pressure therapy or on high-flow nasal cannula oxygen therapy) or for 10 days (while the patient was intubated or on bi-level positive airway pressure therapy or on high-flow nasal cannula oxygen therapy). In addition, low molecular weight heparins (enoxaparin/dalteparin) for anticoagulation were used in patients of the study. In case of patients discharged to home before completing the course of recommended drug therapy, the residual therapy was given to patients at home solely facilitated by the hospital itself. All the patients in the study completed the total 14 days of baricitinib therapy (HD or UD) at the hospital except the patients died within 30-day of study period in both groups.

### Inclusion criteria

Sample inclusion criteria were as follows:

- SARS-CoV 2 is present in the nasal/oral swabs
- no previous history of COVID-19 infection
- having at least two additional signs of severe COVID-19 pneumonia with confirmed pneumonia lesions (bilateral ground-glass opacities) (> 50%) in the chest computerized tomography (CT) scan images at the time of admission: (1) dyspnoea; (II) oxygen saturation in blood (SpO_2_) level ≤ 93% on room air; and (III) respiratory rate ≥ 30 breaths/min
- Onset of symptom(s)-to-hospitalization no more than 10 days
- No history of taking any anti-inflammatory drugs within last 3 months of hospital admission

### Exclusion criteria

Sample exclusion criteria were as follows:

- patient with pregnancy
- any history of trauma or surgical procedure within the last 3 months of hospital admission
- any history of acute/chronic autoimmune disease or active/latent tuberculosis infection
- history of hospital stay for > 3 days for any purpose with the last 3 months
- current evidence of bacterial or fungal coinfection
- coming from another hospital or healthcare facility

### Definition of severe COVID-19 pneumonia

The severe stage of COVID-19 infection in a patient is determined with the evidence of bilateral multi-focal opacities (pneumonia) in the CT-scan images (lung infiltrates > 50% within 24 to 48 h) and other (one or more) signs of disease severity, including dyspnoea, fast breathing rate (≥30 breaths/min), and SpO2 ≤ 93% on room air (RA) [15].

### Data analysis

The statistical analyses were performed by Statistical Product and Service Software (SPSS ver. 22.0, Chicago, IL, USA). Descriptive statistics were presented through median value and interquartile range (IQR). Continuous variables were compared using Mann–Whitney U test, and categorical variables were compared using Pearson Chi-square test. To analyze overall survival in the groups (high dose vs usual dose) we plotted Kaplan-Meier curves. A *P* value of ≤0.05 was considered statistically significant.

## Results

The number of male patients in both the groups was higher than the number of female patients with a median age of 63 (IQR: 54.8–69) and 59 (IQR: 54–68) in HD and UD group, respectively (*P* > 0.05). The median time from onset of symptoms-to-hospitalization was less than 10 days in all patients of this study. With fever [100 (IQR: 100–101)/101 (IQR: 100–101) (*P* = 0.306)], other symptoms, including dry cough (100%/100%), weakness (100%/100%), shortness of breath (78.7%/81%) (*P* = 0.429), anosmia (64.8%/75.9%) (*P* = 0.029), diarrhea (55.7%/49.1%), and sore throat (50.8%/60.3%)) were diagnosed in patients of HD (*N* = 122) and UD group (*N* = 116), respectively. Diabetes, hypertension, cardiovascular disease, bronchial asthma, chronic kidney disease, chronic obstructive pulmonary disease, obesity, peptic ulcer disease, chronic liver diseases, malignancy, and Parkinson’s disease were found as predisposed chronic diseases in patients with COVID-19 of this study (Table 1). Clinical characteristics, including SpO_2_ profile, respiratory and cardiac functions, kidney and liver functions, infection markers, inflammation marker, and hematological components of all the patients in both the groups are given in Table 1 and compared, statistically.

**Table 1:** Baseline demographic information, symptoms of COVID-19, comorbidity and laboratory findings in patients during admission

Variable	HD group (***N*** = 122)	UD group (***N*** = 116)	***P*** value
Male/female, n (%)	83/39 (68/32)	76/40 (66/34)	
Age (year), median (IQR)	63 (54.8–69)	59 (54–68)	0.655
Onset of symptom-to-hospitalization time, median (IQR)	6 (6–8)	7 (6–8)	0.144
Fever (°F), median (IQR)	101 (100–101)	101 (100–101)	0.306
Dry cough, n (%)	122 (100)	116 (100)	
Weakness, n (%)	122 (100)	116 (100)	
Shortness of breath, n (%)	96 (78.7)	94 (81)	0.429
Anosmia, n (%)	79 (64.8)	88 (75.9)	0.029
Diarrhea, n (%)	68 (55.7)	57 (49.1)	0.001
Sore throat, n (%)	62 (50.8)	70 (60.3)	0.001
Diabetes, n (%)	95 (77.9)	88 (75.9)	0.392
Hypertension, n (%)	86 (70.5)	81 (69.8)	0.166
CVD, n (%)	55 (45.1)	47 (40.5)	0.001
Bronchial asthma, n (%)	43 (35.2)	36 (31)	0.001
CKD, n (%)	21 (17.2)	23 (19.8)	0.001
COPD, n (%)	17 (13.9)	14 (12.1)	0.001
Obesity, n (%)	15 (12.3)	14 (12.1)	0.001
PUD, n (%)	11 (9)	16 (13.8)	0.001
CLD, n (%)	6 (4.9)	8 (6.9)	0.001
Malignancy, n (%)	3 (2.5)	4 (3.4)	0.001
PD, n (%)	2 (1.6)	1 (0.9)	0.001
SpO_2_ (%), median (IQR)	90 (88–90.3)	90 (88–90)	0.628
RSO, median (IQR)	7 (4–11)	7 (4–10)	0.640
Respiratory rate, (breaths/min), median (IQR)	25 (21–26)	21 (19–25)	0.001
Heart rate (beat/min), median (IQR)	98 (85–106)	90 (85–102)	0.072
CRP (mg/L), median (IQR)	179 (76.9–263.5)	159 (44.5–231.4)	0.199
Procalcitonin (ng/mL), median (IQR)	1.04 (0.08–2.69)	0.9 (0.12–1.26)	0.001
WBC (K/μL), median (IQR)	9.5 (6.4–12.4)	9.2 (6.7–12.1)	0.915
Neutrophils (%), median (IQR)	86 (77.2–88.9)	86.3 (78.6–90.2)	0.110
Lymphocytes (%), median (IQR)	14.3 (11.5–16.5)	13.9 (10.8–16.4)	0.141
Platelet (K/μL), median (IQR)	188.5 (144.8–256)	215 (150.7–295.8)	0.327
D-dimer (mg /L FEU), median (IQR)	5.2 (3.9–6.8)	4.9 (2.6–6.3)	0.392
IL-6 (pg/mL), median (IQR)	47 (17.5–78)	45 (10.5–75.7)	0.153
Serum Ferritin (ng/mL), median (IQR)	605 (478–786)	641 (456–787.5)	0.144
LDH ((U/L), median (IQR)	489 (409–646.7)	474 (408.5–593)	0.088
Creatinine (mg/dL), median (IQR)	1.1 (0.8–1.9)	1.1 (0.7–1.7)	0.122
ALT (U/L), median (IQR)	58 (46–83)	56 (40.7–75.8)	0.485
AST (U/L), median (IQR)	36.5 (29–48)	35 (29–46)	0.011
MEWS, median (IQR)	3 (2–3)	3 (2–3)	0.001

*IQR* Interquartile range, *n* Number, % Percentage, °*F* Grade Fahrenheit, *CVD* Cardiovascular disease, *CKD* Chronic kidney disease, *COPD* Chronic obstructive pulmonary disease, *PUD* Peptic ulcer disease, *CLD* Chronic liver disease, *PD* Parkinson’s disease, *SpO*_*2*_ Oxygen saturation in blood, *min* Minute, *RSO* Requirement of supplemental oxygen, *CRP* C-reactive protein, *mg* Milligram, *L* Liter, *FEU* Fibrinogen equivalent units, ng Nanogram, *WBC* White blood cells, *K/μL* Thousand cells per micro liter, *IL* Interleukin, *pg/mL* Picograms per milliliter, *LDH* Lactate dehydrogenase, *U/L* Units per liter, *dL* Deciliter, *ALT* Alanine aminotransferase, *AST* Aspartate aminotransferase, *MEWS* Modified Early Warning Score

**Fig. 1:**
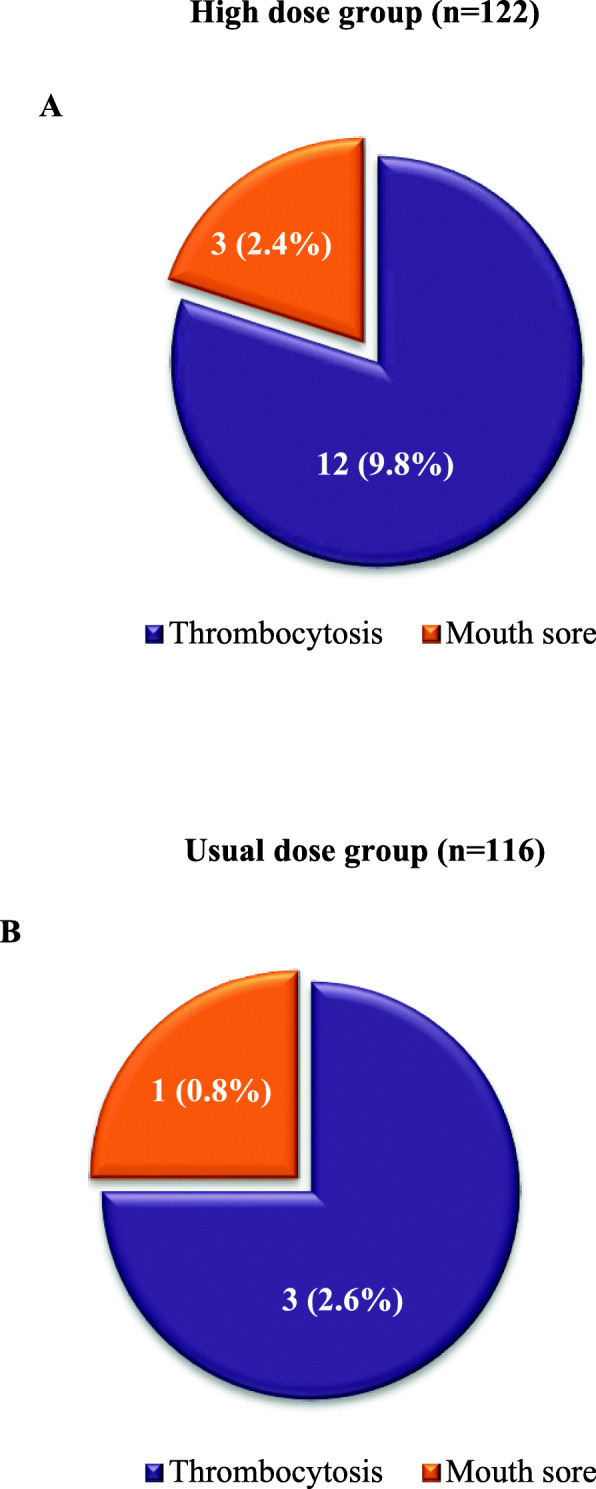
**a** adverse drug events in patients (HD group/case) treated with high dose of baricitinib (*N* = 122), **b** adverse drug events in patients (UD group/control) treated with usual dose of baricitinib (*N* = 116)

Patients in HD group treated with high dose (8 mg) of baricitinib developed thrombocytosis and mouth sore significantly more than the patients received 4 mg usual dose of baricitinib (UD group) (9.8%/2.6 and 2.4%/0.8%, *N* = 122/116, respectively) within the duration of therapy (Fig. 1). Platelet count did not exceed 700 K/μL of blood in any patient of HD or UD group after receiving baricitinib (high or usual dose) and all the patients (*N* = 238) in the study completed the full course (14-day) of baricitinib (high or usual dose) therapy. In clinical justification, no bacterial or fungal or any other opportunistic infections were developed due to baricitinib therapy in patients of both the groups.

**Fig. 2:**
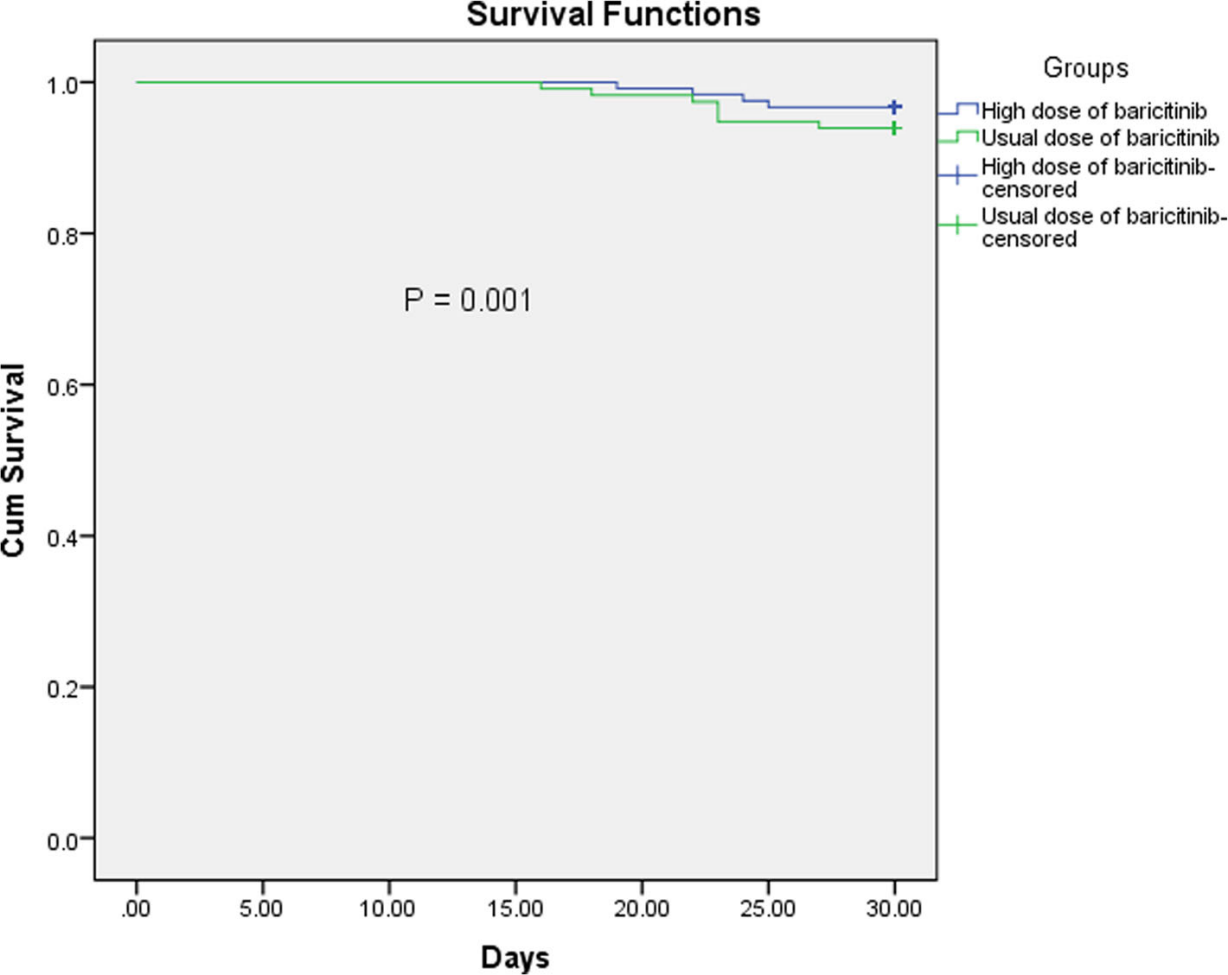
Kaplan-Meier 30-day survival curve for high dose of baricitinib (blue line) and usual dose of baricitinib (green line). Analysis was ran using Group (HD/case vs UD/control) as factor; death as event and time to death as time variable

**Fig. 3:**
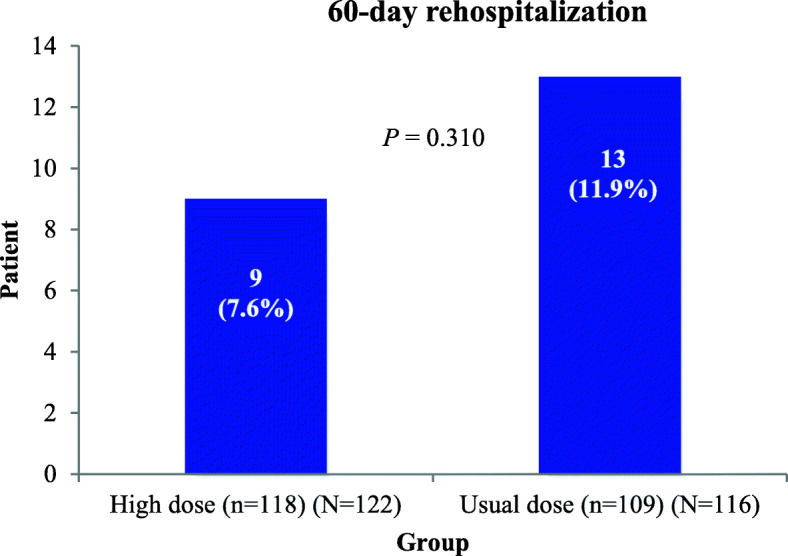
60-day rehospitalization rate of patients with severe COVID-19 infection treated with high (case) or usual (control) oral dose of baricitinib for 14 days

The median day to reach the targeted SpO_2_ (≥94% on RA) was significantly less in the HD group [5 (IQR: 4–5)] than that in the UD group [8 (IQR: 6–9)], and the median day to return in zero supplemental oxygen demand was significantly less in the HD group [5 (IQR: 4–5)] than that in the UD group [8 (IQR: 6–9)]. Nine-percent and 4.1% of patients (*N* = 122) in HD group and 17.2 and 11.2% of patients (*N* = 116) in UD group underwent ICU (*P* = 0.020) and intubation support (*P* = 0.001) due to exacerbation of the disease during their hospitalization time. The median day of hospitalization was lower in the HD group [11 (IQR: 9.5–14)] than that in the UD group [13 (IQR: 10–17.5)] (*P* = 0.072). The 30-day all-cause mortality rate was significantly higher in the usual dose group (6%, *N* = 116) than in the HD group (3.3%, *N* = 122) (Table 2). The Kaplan-Meier 30-day survival curve was analyzed using groups (HD vs UD) and illustrated in Fig. 2. In HD and UD group, 118 (*N* = 122) and 109 (*N* = 116) patients, respectively were discharged to home with acceptable health condition upon medical advice. After discharging from hospital, more patients in UD group were rehospitalized (11.9%, *n* = 109) again due to breathing problems within 60-day of first hospital admission than the patients in HD group (7.6%, *n* = 118) (*P* > 0.05) (Fig. 3).

**Table 2:** Clinical outcomes in patients with severe COVID-19 pneumonia treated with high or usual dose of baricitinib

Parameters	HD group (***n*** = 122)	UD group (***n*** = 116)	***P*** value
Days for SpO_2_ ≥ 94% on room air, median (IQR)	5 (4–5)	8 (6–9)	0.001
Days for no supplemental oxygen demand, median (IQR)	5 (4–5)	8 (6–9)	0.001
Days for respiratory rate < 20 breaths per min	6 (5–6)	8.5 (8–9)	0.001
ICU support, n (%)	11 (9)	20 (17.2)	0.020
Intubation required, n (%)	5 (4.1)	13 (11.2)	0.001
Length of hospital stay (day), median (IQR)	15 (9–18.5)	12 (10–14)	0.072
30-day all-cause mortality, n (%)	4 (3.3)	7 (6)	0.001

*HD* High dose, *UD* Usual dose, *SpO*_*2*_ Peripheral capillary oxygen saturation, *IQR* Interquartile range, *ICU* Intensive care unit, *n* Number, % Percentage

## Discussion

In this study, patients with severe COVID-19 pneumonia treated with high oral dose (8 mg in two divided doses, daily) of baricitinib (started at early hours of hospitalization) showed early stabilization of the respiratory functions (SpO_2_ ≥ 94% on RA and no requirement of supplemental oxygen), lower risk of ICU and intubation support due to exacerbation of the severity of the disease, and length of hospital stay compared to patients treated with usual dose (4 mg once daily) of baricitinib for a period of 14 days. In our recent case-control study on 37 patients with moderate-to-severe COVID-19 pneumonia, we found that compared to usual daily dose regimen (4 mg) of baricitinib, a single 8 mg oral loading dose-based daily usual dose (4 mg) regimen of baricitinib for 14 days revealed better clinical outcomes, including early normalization of SpO_2_ (≥95%) on RA and restoration of normal breathing function [4 (IQR: 4–5)/3 (IQR: 2–8), *P* > 0.05; 8 (IQR: 7–10)/ 5 (IQR: 4–5), *P* < 0.05, respectively] [13].

Several studies reported that among the hospital admitted patients with COVID-19, 15.7 to 26.1% cases are severe and often difficult to clinically justify on the basis of abnormal laboratory investigations and CT scan report [16–18]. Though, there is no standard anti-inflammatory drug therapy for the treatment of severe pneumonia in COVID-19 infection, so early diagnosis and initiation of treatment upon hospital admission with the available drugs may attribute favorable clinical outcome [12, 19]. In this study, along with the antiviral and steroid therapy, the anti-inflammatory drug baricitinib was started in both the groups’ patients with severe COVID-19 pneumonia within 4 h of hospital admission.

In severe state of COVID-19 infection, the activated polyfunctional CD4+ and CD8+ T lymphocytes make the biggest defense against the coronavirus and decreasing their count results in lymphopenia in about 85% of patients with severe COVID-19 infection [20]. In response to reduced T cell count, excessive amount of proinflammatory cytokines (IFN-γ, IL-1, IL-6, IL-12, and TGFβ) are released along with upregulation of chemokines (CCL2, CXCL10, CXCL9, and IL-8). This hyperstimulated inflammatory cascade triggers up the cytokine storm which may ultimately results in acute lung injury (ALI) and acute respiratory distress syndrome (ARDS) leading to respiratory failure [5, 6]. The oral JAK1/JAK2 inhibitor baricitinib has been intended to use as an anti-inflammatory or anti-cytokine agent in COVID-19 in a few setups across the world [12, 13].

A recent in vitro pharmacology study showed that baricitinib selectively inhibits cytokine-dependent inhibition of phosphorylated STAT, a group of transcription factors that are highly regulated by JAK/STAT signaling pathway, following a concentration versus response curve. This baricitinib-driven blockage in cytokine-mediated JAK/STAT signaling pathway potentially leads to reduction in the concentration of L-2, IL-6, IL-10, IFN-γ, and G-CSF, with lower IC (inhibitory concentration) 50 values [21]. The systemic exposure of orally taken baricitinib is dose-dependent [12], and in healthy human volunteers, following oral administration of baricitinib, the peak plasma concentration attained within 1.5 h, pharmacodynamics shows linear dose versus time invariant, accumulates minimally in blood following repeat oral dosing, and pharmacokinetics is directly proportional to its systemic concentration [22]. In our study, 8 mg (4 mg at 12 h interval) oral daily dose of baricitinib (HD group) showed better suppression of pro-inflammatory cytokine release in patients with severe COVID-19 pneumonia with higher systemic drug concentration, and as a result, total breathing function was restored earlier with a reduction in ICU and intubation support compared to the patients in UD group treated with 4 mg orally daily [9%/17.2%, *N* = 122/116, *P* < 0.05; 4.1%/11.2%, *N* = 122/116, *P* > 0.05, respectively]. Antiviral role of baricitinib is an additional benefit of bariticitinib therapy, and study mentioned that in combination with antiviral remdesivir, baricitinib may reduce viral load and strongly aberrant host inflammatory response in COVID-19 [23]. In our study, we used baricitinib with remdesivir (5 days) and steroid (14 days) in both the groups.

Multiple studies reported that massive systemic inflammation and multiple organ failure are the major causes of high hospital mortality rate in patients with severe COVID-19 pneumonia. Respiratory failure (41.6%) was the most common in severe COVID-19 infection followed by acute myocardial infarction (38.9%), acute liver injury (25.7%), and acute kidney injury (23.0%) [14, 17, 18, 24]. A study found that early initiation of baricitinib-based antiviral therapy reduced both the ICU admission and mortality in patients with moderate COVID-19 pneumonia [12]. Studies showed that among the post-COVID-19 patients who were discharged to home, 3.6 to 4.4% of patients readmitted in hospital where the most common cause was respiratory distress (50%) [25, 26]. In this study, 8 mg of oral baricitinib in two-divided dosages (12 h apart) daily in severe COVID-19 cases reduced 30-day mortality, significantly compared to 4 mg once daily dosing regimen [3.3% (*N* = 122)/6% (*N* = 116), respectively], and patients treated with high dose of baricitinib for 14 days (HD group) readmitted to hospital within 60-day of first hospitalization with positive COVID-19 symptoms with breathing problem less than the patients treated with usual dose of baricitinib (UD group) [7.6% (*N* = 122)/11.9% (*N* = 116), *P* > 0.05].

Baricitinib is well tolerated in patients with COVID-19 and associated complications are very rare as mentioned in recent studies [12, 27]. In this study, adverse events (AEs) associated with prescribed medications including baricitinib were monitored and recorded continuously, and 9.8 and 2.4% of patients in HD group (*N* = 122) developed baricitinib-induced thrombocytosis and mouth sore, respectively which was less in the patients of UD group (thrombocytosis/mouth sore: 2.6 and 0.8%, respectively). But these AEs were not serious or life-threatening. No patient in the study (HD/UD group) experienced an elevated platelet count above 700 K/μL of blood during hospital stay and all the patients completed 14-day long baricitinib therapy. Mouth sore was healed with the application of amlexanox 50 mg/g oral paste four times a day. Study found that baricitinib at an 8-fold higher systemic concentration attained after a 4 mg dose does not cause hepatic cell damage [21]. However, the 14-day duration of the baricitinib therapy was not reduced in any group (HD or UD) of this study due to the development of AEs. Because a recent study highlighted that recurrent SARSCov-2 was detected in nasopharyngeal swabs in rapidly recovered and discharged-to-home patients treated with a 10-day long baricitinib therapy [21].

The recent EUA from US FDA regarding the use of baricitinib in COVID-19 is a new hope for frontline clinicians around the globe in order to save their patients from hyperinflammation and other serious complications in-advance associated with moderate-to-severe severe COVID-19 infections [10]. Soon, some randomized controlled studies are highly required to make evidence the benifits of using high dose of baricitinib in severe COVID-19 pneumonia rather than usual dose. The major limitations of this study were: single-center study on same origin of population (South Asian), small sample size, non blinded study, and no pharmacokinetics study to determine the systemic drug exposure level.

## Conclusion

Hyperinflammatory response-associated serious complications leading to multiple organ failures are life-threatening situation in severe COVID-19 infection. Baricitinib has been spotlighted as a potential anti-inflammatory agent in COVID-19 infection. This study found that an 8 mg daily oral dose of baricitinib for 14-day revealed early normalization of respiratory function, reduced need of ICU and intubation support, declined 30-day mortality rate, and minimized 60-day rehospitalization rate compared to usual 4 mg daily oral dose of baricitinib in severe COVID-19 pneumonia.

## Data Availability

Study data are available upon request to corresponding author.

## Abbreviations

COVID-19: Coronavirus disease 2019
SARS-CoV-2: Severe acute respiratory syndrome coronavirus 2
WHO: World Health Organization
ACE2: Angiotensin-converting enzyme 2
IL-6: Interleukin-6
MCP1: Monocyte chemoattractant protein
MIP1A: Macrophage inflammatory protein
TNF-alpha: Tumor necrosis factor
IP10: Interferon gamma inducible protein
CCL-2: CC-chemokine ligand 2
CXCL-10: CXC-chemokine ligand 10
MNP: Mononuclear phagocyte
FDA: Food and Drug Administration
EUA: Emergency Use Authorization
JAK: Janus kinases
STATs: Signal transducers and activators of transcription proteins
ACTT-2: Adaptive COVID-19 Treatment Trial 2
CI: Confidence interval
SCU: Specialized COVID-19 Unit
RT-PCR: Reverse transcriptase polymerase chain reaction
HD: High dose
UD: Usual dose
CT: Computerized tomography
SpO_2_: Oxygen saturation
RA: Room air
SPSS: Statistical product and service software
IQR: Interquartile range
ALI: Acute lung injury
ARDS: Acute respiratory distress syndrome
IC: Inhibitory concentration
AE: Adverse event
